# 

**Published:** 1897-09

**Authors:** 


					﻿Vol. XVII.
SEPTEMBER, 1097.
NO. 9?
THE
pOMCEOPATHlC pHY^lClAft
A Monthly Journal of Homoeopathic Materia Medica
and Clinical Medicine.
•* He is a freeman whom truth makes free, and. all are slaves beside."
"Seek the Truth: come whence it may, cost what it will."
BDITBD AND PUBLISHED BY
WALTER Tvf. JAMES, M. D.
PHILADELPHIA:
Ko. 1231 LOCUST STREET.
X. o jt 0 rit :
ALFRED HEATH & CO., Sole Agents for Great Britain,
114 ■Ebury Street, Eaton Square.
•Yearly Subscription, $2.50, invariably in advance. Single numbers, 25 cts.
Entered at the Philadelphia Poit-Office a. Seoond-Claae Mail Matter.
				

